# Performance Comparison of Solid Lead Ion Electrodes with Different Carbon-Based Nanomaterials as Electron-Ion Exchangers

**DOI:** 10.3390/s21051663

**Published:** 2021-02-28

**Authors:** Lei Zhang, Zhengying Wei, Pengcheng Liu, Haoran Wei, Denglong Ma

**Affiliations:** State Key Lab for Manufacturing System Engineering, Xi’an Jiaotong University, Xi’an 710049, China; zywei@mail.xjtu.edu.cn (Z.W.); jerryz2017@stu.xjtu.edu.cn (P.L.); weihaoran@stu.xjtu.edu.cn (H.W.); denglong.ma@xjtu.edu.cn (D.M.)

**Keywords:** Pb^2+^ selective electrode, graphene, multi-walled carbon nanotube, fullerene

## Abstract

Carbon-based nanomaterials with carboxylation or chemical modification are widely used as electron-ion exchangers of solid electrodes. For reducing the complexity and dangerousness of the intermediate layer preparation, different original carbon-based nanomaterials are dispersed in deionized water. They are applied in the fabrication of Pb^2+^-selective electrodes. Because the contact angle of graphene reached 132.5°, the Pb^2+^-selective electrode of graphene used as an electron-ion exchanger showed excellent performance with a low detection limit of 3.4 × 10^−8^ M and a fast average response time of 42.6 s. The Nernstian response slope could reach 26.8 mV/decade, and the lifetime lasted for a month. Therefore, graphene suspension without any treatment can be used as the intermediate layer of solid-state electrodes, providing a reference for the preparation of other ion-selective electrodes.

## 1. Introduction

Heavy metals such as lead increasingly aggravate the pollution of ecosystems and the poisoning of organisms [1]. Therefore, it is necessary to monitor the lead content [2]. There are many kinds of techniques for detection and the typical analytical methods include atomic absorption spectrometry (AAS) [3], ultraviolet-visible spectrophotometer [4], high performance liquid chromatography (HPLC) [5] and X-ray fluorescence (XRFS) [6], which exhibit disadvantages such as complex operation, poor portability and expensive instruments. The ion-selective electrodes, especially the all-solid-state ion-selective electrodes (ASS-ISEs), are widely applied in environmental monitoring [7,8,9,10] owing to portability, low cost and simple operation [11].

**Figure 1:**
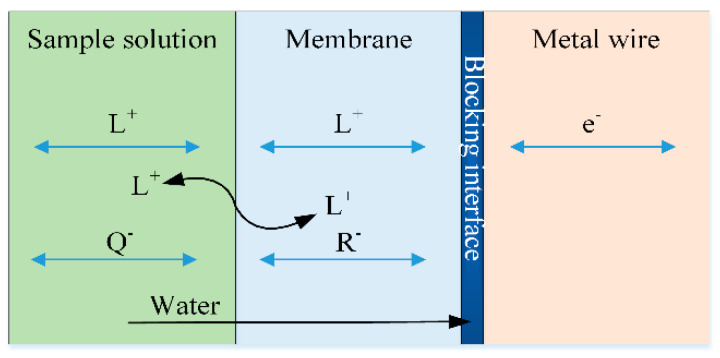
Charge transportation and blocking interface in coated wire electrodes (CWE). L^+^: cation to be measured; Q^−^: anion in sample solution, R^−^: anion in membrane; e^−^: electron.

The coated wire electrodes (CWEs), in which a thin polymeric film comprising electroactive ionophore is dip-coated directed on the metal wire [12], are the initial formation of ASS-ISEs. Although the preparation of CWEs is simple, the potentials drift chaotically because of the “blocking” membrane/wire interfaces (Figure 1) and the sensitivity to small accidental charges [13]. The water may penetrate through the sensitive membrane, then a thin water layer is formed on the wire surface. The instability of electrode potentials is caused by the unstable metal ion concentration in the aqueous layer. Therefore, a great deal of research has been focused on the use of an electron-ion exchanger to improve the “blocking” interfaces and electrode performance. Due to high conductivity [14,15], conductive polymers (CPs) are employed as intermediate layers in solid-state ISEs [16]. For instance, the commercially available CPs named polyaniline (PANI) was drop-casted on acetate transparency sheets [17] and another applicable CPs called poly(3,4-ethylenedioxythiophene) (PEDOT) were electrochemically deposited onto magnesium microwires [18]. After drying, the cocktails of ion-selective membrane were coated on the CPs layer. The introduction of CPs as an electron-ion exchanger improved the potential stability. However, the formation of a thin water film between the CPs and ion-sensitive membrane resulted in chemical hysteresis [19]. Moreover, the oxidation reaction with dissolved oxygen limited the performance of the CPs-based electrode.

Due to the good hydrophobicity and conductivity, carbon-based nanomaterials are widely exploited as electron-ion exchangers instead of CPs. Graphene (GR) (Figure S1a) is a two-dimensional crystal composed of closely packed carbon atoms. It has high strength, good toughness, light weight, high light transmittance, good conductivity and large specific surface area. The main tube of carbon nanotubes (CNTs) (Figure S1b) is formed by curling a part of GR sheets. Fullerene (C_60_) (Figure S1c) is obtained by bending a part of GR into a football shape. Therefore, three carbon-based nanomaterials have been extensively studied and applied to sensors. Potentiometric sensors based on multi-walled carbon nanotube (MWCNT) as inner transducing layer exhibited good resistance to potential drift and the formation of water films [20,21,22]. C_60_, an efficient charge-transfer mediator, was used as solid contact layer for ASS-ISEs. The characteristics of the electrode showed that the ion-to-electron transduction and the potential stability were promoted [23,24]. Amr M. Mahmoud fabricated an eco-friendly solid-contact selective electrode with GR as an electron-ion exchanger for the determination of flavoxate hydrochloride; the prepared electrode showed little potential drift and good stability in comparison with the electrode without the graphene layer [25]. Surface groups were produced in the carbon-based nanomaterials by purification, carboxylation and chemical modification [22,25,26,27,28]. The carboxylated and modified nanomaterials were suitable as substrates for reacting with specific ions, but the structural damage would reduce their mechanical properties. At the same time, the complexity and dangerousness of material preparation increased. In order to solve these problems, GR, MWCNT and C_60_ selected were dispersed in the deionized water (DI water), respectively. The suspension was drop-cast on substrate electrode, followed by natural drying to form the electron-ion exchanger. The optimal solid Pb^2+^-ISE was obtained by comparing the properties of three electrodes with different carbon-based nanomaterials as the intermediate layer.

## 2. Experimental

### 2.1. Reagents and Materials

Glassy carbon (GC) electrodes were purchased from Xuzhou Zhenghao Electronics Co., Ltd. (Xuzhou, China). Reagents including o-nitrophenyl octyl ether (o-NPOE), poly(vinyl chloride) (PVC), anhydrous ethanol and lead nitrate (Pb(NO_3_)_2_) standard solution were obtained from Shanghai Aladdin Industrial Corporation (Shanghai, China). Lead ionophore IV (Figure S2), tetrahydrofuran (THF) and acetone were purchased from Tianjin Kemiou Chemical Reagent Co., Ltd. (Tianjin, China). Potassium chloride (KCl, >99.5%), sodium tetrakis[3,5-bis(trifluoromethyl)phenyl]borate (NaTFPB) and potassium ferricyanide (K_3_[Fe(CN)_6_]) of analytical grade were acquired from Tianjin Tianli Chemical Reagent Co. (Tianjin, China). GR. MWCNT and C_60_ were provided by Nanjing Ji Cang Nano Technology Co., Ltd. (Nanjing, China), Beijing Tsinghua-Nafine Nano-Powder Commercialization Engineering Center and Nanjing Xianfeng Nano Material Technology Co., Ltd. (Nanjing, China), respectively. The corresponding solutions were prepared in DI water (resistance 18.25 MΩ·cm, ULUPURE, Chengdu, China).

### 2.2. ISEs Fabrication

The glassy carbon (GC) electrodes with a diameter of 3 mm were polished in aqueous dispersions of alumina powder (0.5, 0.3 and 0.05 μm, respectively). Then, the GC electrodes were ultrasonically cleaned in DI water and anhydrous ethanol for 3 min separately and dried under nitrogen. Finally, the cyclic voltammetry characteristics of GC electrodes were measured in K_3_[Fe(CN)_6_] solution to obtain a qualified substrate electrode pretreated (in Section 2.3). Carbon-based nanomaterial (GR, MWCNT and C_60_, respectively) was dispersed by ultrasonic vibration in DI water for 12 h to form a 1 mg/mL uniform suspension. The electron-ion exchanger was acquired by drop casting 50 μL carbon-based nanomaterial dispersion on the qualified GC electrodes, followed by drying at room temperature (yielding GC/GR, GC/MWCNT and GC/C_60_ electrodes). The Pb^2+^ ion-selective membrane cocktail (total mass 250 mg) was prepared by dissolving lead ionophore IV (1.4 wt%), NaTFPB (0.6 wt%), plasticizer (63 wt%, o-NPOE) and PVC (35 wt%) in 2 mL THF. Subsequently, 20 μL cocktail was drop-cast onto the GC/GR, GC/MWCNT and GC/C60 electrodes evenly and the solvent was evaporated thoroughly at room temperature (producing GC/GR/Pb^2+^-ISE, GC/MWCNT/Pb^2+^-ISE and GC/C_60_/Pb^2+^-ISE).

For the measurements, the fabricated electrodes were conditioned in 10^−3^ M Pb(NO_3_)_2_ solution for at least 12 h and then in 10^−9^ M Pb(NO_3_)_2_ solution for more than 24 h.

### 2.3. Apparatus and Measurements

The morphologies of GR, MWCNT and C_60_ suspension were tested using field emission scanning electron microscope (FESEM) (SU8010, Hitachi, Tokyo, Japan).

Contact angle (CA) measurements were determined with an optical contact angle measuring instrument (DSA100, KRUSS, Hamburg, German). Approximately 2 μL DI water was placed on the electron-ion exchanger formed by different nanomaterials. CA values were calculated automatically by the equipment matching software.

The cyclic voltammograms and potential responses were measured with an electrochemical workstation (PARSTAT3000, Ametek, Berwyn, PA, USA) in a three-electrode system: working electrode—GC/GR/Pb^2+^-ISE (GC/MWCNT/Pb^2+^-ISE or GC/C60/Pb^2+^-ISE); reference electrode—Hg/Hg_2_Cl_2_/saturated KCl electrode (SCE); counter electrode—platinum wire electrode. The pretreated GC electrodes were tested by cyclic voltammetry in the potential range from −0.1 V to 0.6 V at a constant scan rate of 50 mV/s in 1 mM K_3_[Fe(CN)_6_] solution. The potential difference (<80 mV) between oxidation peak and reduction peak is a criterion for judging the qualification of the pretreatment. The stability of Pb^2+^-ISEs were measured by cyclic voltammetry (scan potential −0.5 V–0.5 V, scan rate 50 mV/s, scan circle 20) in a 0.1 M KCl solution. The calibration curves of electromotive force (EMF) were obtained in the gradient Pb(NO_3_)_2_ solutions from low to high concentrations (10^−11^ to 10^−3^ M).

## 3. Results and Discussions

### 3.1. Micrograph of ElectronIon Exchanger

**Figure 2:**
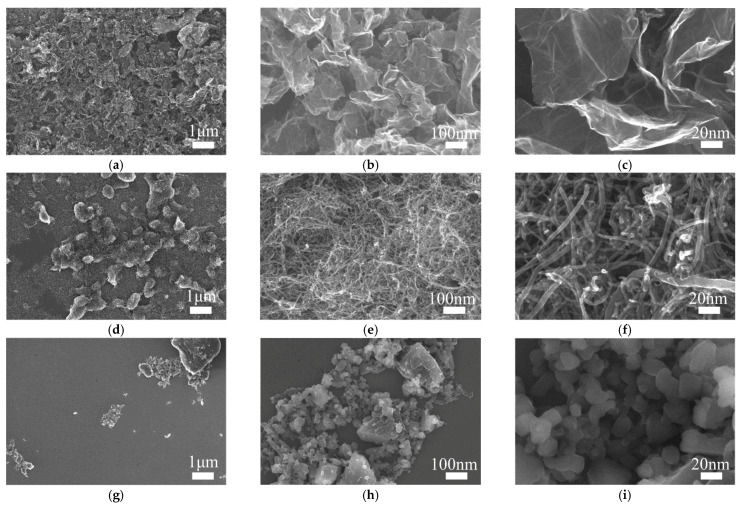
SEM images of the carbon-based nanomaterials at different magnifications. (**a**) Graphene (GR) × 1 K. (**b**) GR × 10 K. (**c**) GR × 50 K. (**d**) Multi-walled carbon nanotube (MWCNT) × 1 K. (**e**) MWCNT × 10 K. (**f**) MWCNT × 50 K. (**g**) C_60_ × 1 K. (**h**) C_60_ × 10 K. (**i**) C_60_ × 50 K.

Figure 2 shows the SEM im-ages of different carbon-based nanomaterials used as the electron-ion exchanger. At 1 K magnification, C_60_ are only distributed in a small area, and the size difference is obvious (Figure 2g) for the reason that the dispersibility of C_60_ in anhydrous ethanol is very bad. At the same magnification, GR is more evenly distributed and has a more consistent size (Figure 2a) than MWCNT (Figure 2d). As shown in Figure 2b,c,e,f,h,i, flake GR is stacked and distributed in the conducting layer uniformly, MWCNT is aggregated seriously and entwined and C_60_ is distributed in clumps. Therefore, suspension of GR is more suitable for fabricating the electron-ion exchanger.

### 3.2. CA of Carbon-Based Nanomaterials

Wettability [29] is the capacity of the liquid to spread on a solid surface. CA is an important method to characterize the wettability. The solid-liquid CA (Figure S3) is the angle between the boundary tangent of a droplet and a solid surface. An intermediate layer (electron-ion exchanger) introduced between the sensitive membrane and the substrate electrode plays a significant role in inhibiting the formation of the water layer. Hence, the CA of electron-ion exchanger directly affects the shielding effect for the water layer. The 90° CA is the critical point between hydrophilicity and hydrophobicity. If the CA is more than 90°, the hydrophobicity is better.

**Figure 3:**
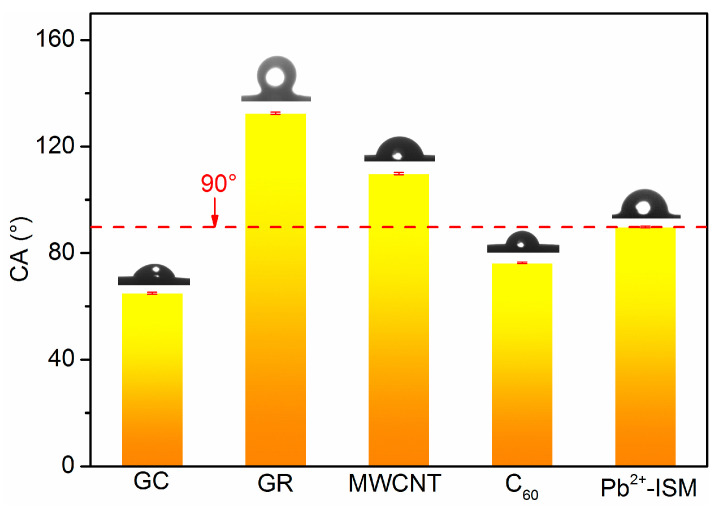
Contact angle (CA) of different carbon-based nanomaterials intermediate layer.

As indicated in Figure 3, the CA of GC is 64.9 ± 0.4° and the water layer is formed easily because of the high hydrophilicity. The CA of the Pb^2+^ ion-sensitive membrane (Pb^2+^-ISM) is close to 90°. Without a hydrophobic electron-ion exchanger, the performance of the electrode will be affected. GR, MWCNT and C_60_ electron-ion exchanger were prepared, respectively, then their CAs were measured. Figure 3 shows that the CA of the C_60_ electron-ion exchanger is larger than that of GC. However, the CA is 76.3 ± 0.3° (less than 90°) and the C_60_ intermediate layer is manifested as hydrophilicity. It is consistent with the results of micrograph. The CAs of GR and MWCNT are more than 90° and their electron-ion exchangers show hydrophobicity. The CA in MWCNT electron-ion exchanger is 109.8 ± 0.4°, so its hydrophobicity is weak. The CA of GR is 132.5 ± 0.4° and the hydrophobicity is optimal.

### 3.3. Potentiometric Performance

**Figure 4:**
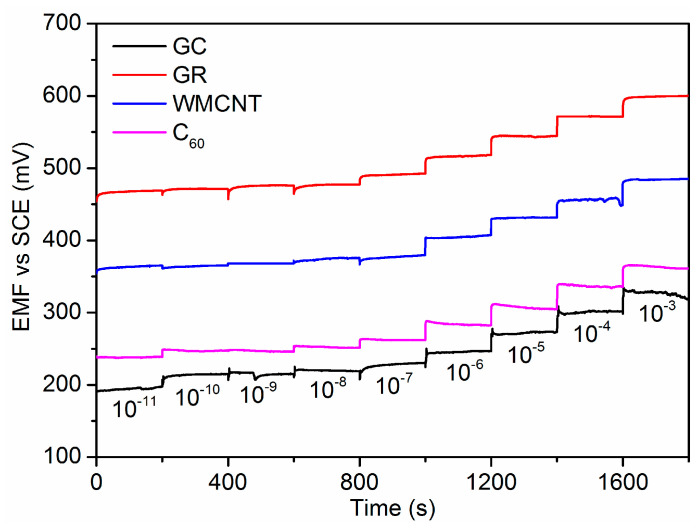
Potentiometric response of the different Pb^2+^-ion-selective electrodes (ISEs) with lead concentrations from 10^−11^ to 10^−3^ M.

**Figure 5:**
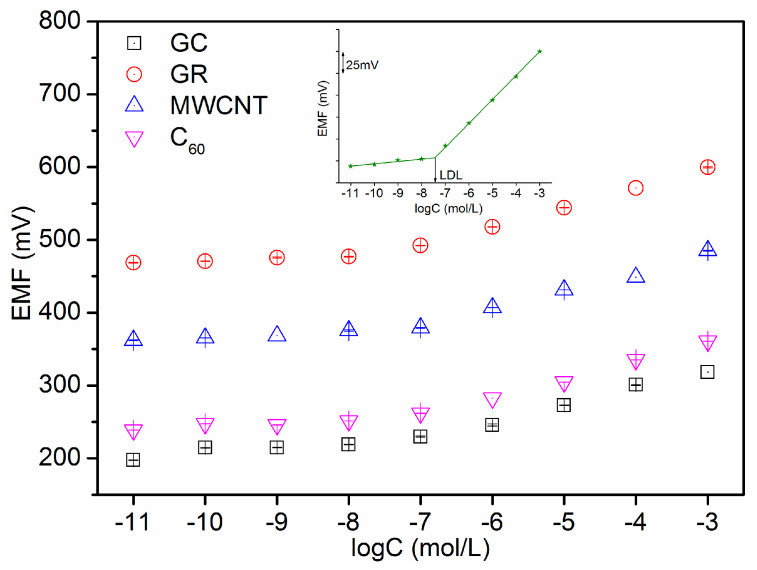
Potentiometric calibration curve of different Pb^2+^-ISEs in lead concentration range from 10^−11^ to 10^−3^ M.

**Figure 6:**
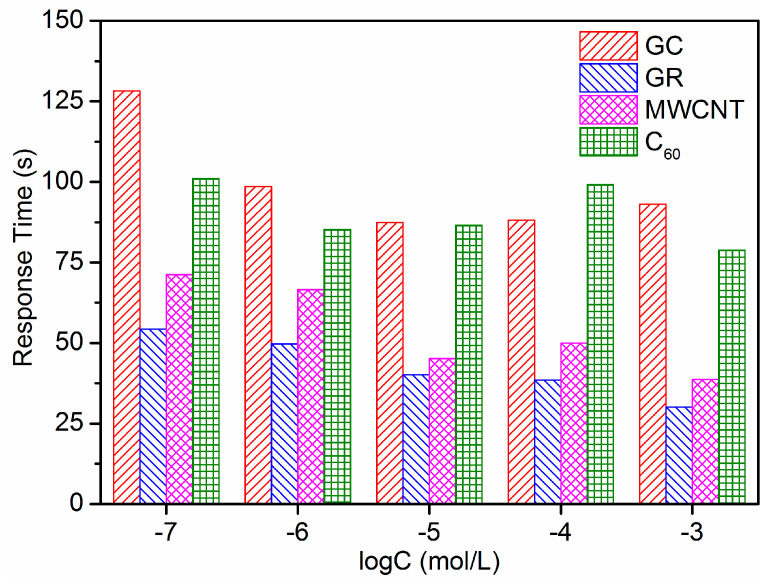
Potentiometric response time of different Pb^2+^-ISEs in the range of Nernstian response.

The potentiometric responses of Pb^2+^-ISE containing different carbon-based nanomaterials electron-ion exchangers were investigated in the range of 10^−11^ to 10^−3^ M. As shown in Figure 4, all ISEs are insensitive to the change of low Pb(NO_3_)_2_ solution concentrations (below 10^−8^ M). However, the evident EMF step-up change is observed with the increase in Pb^2+^ concentration at high concentrations (e.g., above 10^−7^ M). Figure 5 demonstrates that the proposed electrodes show a Nernstian response over a wide range of 10^−7^ to 10^−3^ M. The low detection limit (LDL) was calculated as the intersection of the two liner lines (the inset in Figure 5). The Nernstian slope and LDL of four electrodes were recorded in Table 1. It can be seen that the characteristics of the electrodes with carbon-based nanomaterials electron-ion exchangers are superior to those of the electrode without an electron-ion exchanger. The GC/GR/Pb^2+^-ISE shows a Nernstian response of 26.8 ± 0.3 mV/decade over a wide range of 10^−7^ to 10^−3^ M. The LDL calculated is 3.4 × 10^−8^ mol/L. Response times of all ISEs in the range of Nernstian response are compared in Figure 6. The GR electron-ion exchanger produced less response time. Compared with the response time of GC/Pb^2+^-ISE, the average response time of GC/GR/Pb^2+^-ISE was reduced by 57%. The value was 42.6 s. These results proved the hydrophobicity was GR > MWCNT > C_60_, which was consistent with the tests of CA. The potentiometric performance of GC/GR/Pb^2+^-ISE was the best. The performance of GC/GR/Pb^2+^-ISE with different thickness of Pb^2+^-ISM is shown in Table S1.

### 3.4. Stability and Conductivity of ISEs

**Figure 7:**
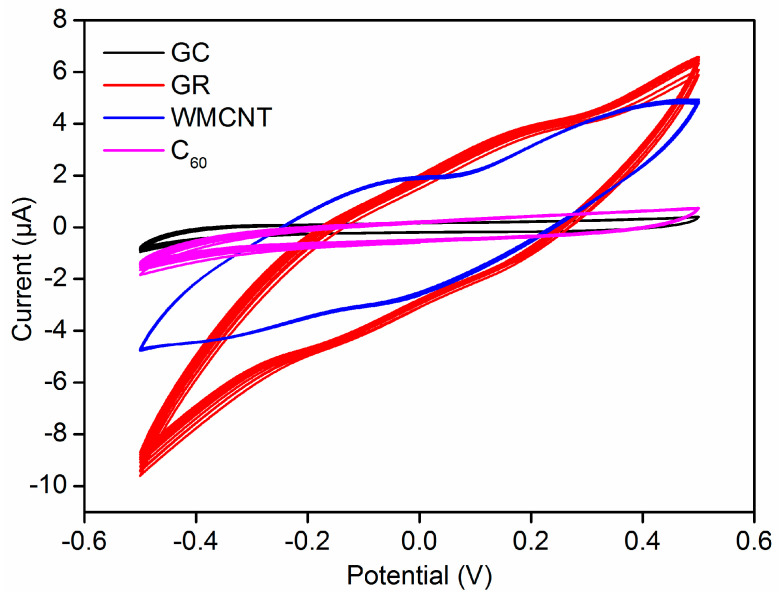
Multi-circle cyclic voltammetry curves of different Pb^2+^-ISEs.

In the practical measurements, the stability of ISE has great influence on the accuracy due to the drift of potential. Multi-circle cyclic voltammetry scan was performed in a 0.1 M KCl solution. The 20-circle scan curves of each electrode have only slight offset as shown in Figure 7; hence, the prepared electrodes display good stability. In Section 2.2, the carbon-based nanomaterials dispersion is easy to prepare. However, there are some defects in the dispersion uniformity, which are also consistent with the morphologies of SEM. In the 10^−5^ mol/L lead solution, GC/GR/Pb^2+^-ISE and GC/MWCNT/Pb^2+^-ISE were tested for 3000 s, respectively. The potentials remain stable as shown in Figure S4. The currents of GC/GR/Pb^2+^-ISE and GC/MWCNT/Pb^2+^-ISE are higher than those obtained at GC/C_60_/Pb^2+^-ISE and GC/Pb^2+^-ISE because GR and MWCNT have a large electron-transfer ability and high specific surface area. Furthermore, the lead ion electrode with GR as the intermediate layer has the best conductivity.

### 3.5. Lifetime of ISEs

**Figure 8:**
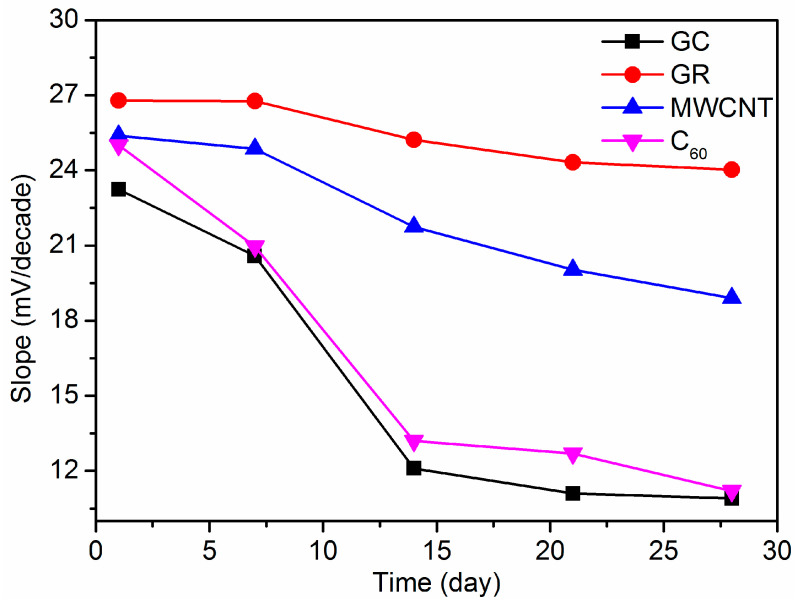
Long-time slope of different Pb^2+^-ISEs.

The long-period potential responses of the fabricated electrodes were tested to obtain the Nernstian slope in the range of 10^−7^ m to 10^−3^ M Pb^2+^ solution. As shown in Figure 8, the response slopes of GC/C_60_/Pb^2+^-ISE and GC/Pb^2+^-ISE decrease by half rapidly after two weeks. No obvious loss of response slopes is observed within a week for GC/GR/Pb^2+^-ISE and GC/MWCNT/Pb^2+^-ISE. After 28 days, the response slopes of GC/GR/Pb^2+^-ISE and GC/MWCNT/Pb^2+^-ISE reduce by 10.3% and 25.6%, respectively. However, the electrode with GR as intermediate layer still presents a Nernstian response slope of more than 24 mV/decade. The robust and reliable GC/GR/Pb^2+^-ISE is promising for applications in many fields such as drinking water and industrial wastewater. When the prepared electrodes are not in use, they should be dipped in the conditioning solution of 10^−9^ M Pb(NO_3_)_2_.

### 3.6. Comprehensive Property and Follow-up Studies

The properties of the four electrodes manufactured in this paper are compared with those in the literatures. As shown in Table 2, five indexes including linear range, slope, LDL, response time and lifetime represent the performances of electrodes. The Pb^2+^ electrode with GR as electron–ion exchanger has better comprehensive performance and is suitable for testing a Pb^2+^ solution of low concentration.

In addition, the slope, response time and lifetime of GC/GR/Pb^2+^-ISE can be improved further for extensive application.

## 4. Conclusions

This study demonstrated that different carbon-based nanomaterials can be applied in the fabrication of Pb^2+^-selective electrodes. Especially, the Pb^2+^-selective electrode of graphene used as an electron-ion exchanger showed excellent performance with a low detection limit of 3.4 × 10^−8^ M and a fast average response time of 42.6 s. The Nernstian response slope could reach 26.8 mV/decade. The addition of a graphene intermediate layer in the Pb^2+^-selective electrode enhanced the hydrophobicity which was testified by contact angle measurement. Moreover, the GC/GR/Pb^2+^-ISE displayed good potential stability and conductivity. Furthermore, the lifetime of the electrode lasted for a month. When the graphene suspension was fabricated for the electron-ion exchanger of Pb^2+^-selective electrode, the potential performance and lifetime could be improved. Therefore, graphene can be used as the intermediate layer of solid-state electrodes, providing a reference for the preparation of other ion-selective electrodes.

## Figures and Tables

**Table 1:** Comparison of different Pb^2+^-ISEs characteristics.

ISE	Slope (mV/Decade)	LDL (mol/L)
GC/Pb^2+^-ISE	23.2 ± 1.3	1.1 × 10^−7^
GC/GR/Pb^2+^-ISE	26.8 ± 0.3	3.4 × 10^−8^
GC/MWCNT/Pb^2+^-ISE	25.4 ± 1.5	8.7 × 10^−8^
GC/C_60_/Pb^2+^-ISE	25.0 ± 1.1	6.4 × 10^−8^

**Table 2:** Comparison of comprehensive performance for different Pb^2+^ electrodes.

No.	Linear Range (mol/L)	Slope (mV/decade)	LDL (mol/L)	Response Time (s)	Lifetime (day)	Note
1	10^−7^~10^−3^	23.24	1.10 × 10^−7^	99.12	1	GC/Pb^2+^-ISE
2	10^−7^~10^−3^	26.79	3.44 × 10^−8^	42.58	28	GC/GR/Pb^2+^-ISE
3	10^−7^~10^−3^	25.39	8.65 × 10^−8^	54.34	7	GC/MWCNT/Pb^2+^-ISE
4	10^−7^~10^−3^	25.01	6.39 × 10^−8^	90.22	1	GC/C_60_/Pb^2+^-ISE
5	10^−5^~10^−1^	26	-	60	14	[30]

## Data Availability

All data included in this study are available upon request by contact with the corresponding author.

## Supplementary Materials

The following are available online at https://www.mdpi.com/1424-8220/21/5/1663/s1, Figure S1: Schematic of carbon-based nanomaterial structure, Figure S2: The structure of the lead ionophore IV, 4-tert-butylcalix[4]arene-tetrakis(N,N-dimethylthioacetamide), Figure S3: Schematic of a sessile-drop contact angle system, Table S1: Comparison of GC/GR/Pb^2+^-ISEs with different volume of Pb^2+^-ISM characteristics, Figure S4: EMF of GC/GR/Pb^2+^-ISE and GC/MWCNT/Pb^2+^-ISE in 10^−5^ mol/L lead solution.
