# Supplementary material for: Performance Comparison of Solid Lead Ion Electrodes with Different Carbon-Based Nanomaterials as Electron-Ion Exchangers
Source: Sensors (Basel). 2021 Feb 28;21(5):1663. doi: 10.3390/s21051663 (PMC7957766; doi:10.3390/s21051663)
Supplement: Supplementary file 1 [file sensors-21-01663-s001.pdf]

*Communication*

# Performance Comparison of Solid Lead Ion Electrodes with Different Carbon-Based Nanomaterials as Electron-Ion Exchangers

**Lei Zhang [1,*], Zhengying Wei [1], Pengcheng Liu [1], Haoran Wei [1] and Denglong Ma [1]**

State Key Lab for Manufacturing System Engineering, Xi'an Jiaotong University, Xi'an 710049, China; zywei@mail.xjtu.edu.cn (Z.W.); jerryz2017@stu.xjtu.edu.cn (P.L.); weihaoran@stu.xjtu.edu.cn (H.W.); denglong.ma@xjtu.edu.cn (D.M.)

* Correspondence: zl870127@stu.xjtu.edu.cn

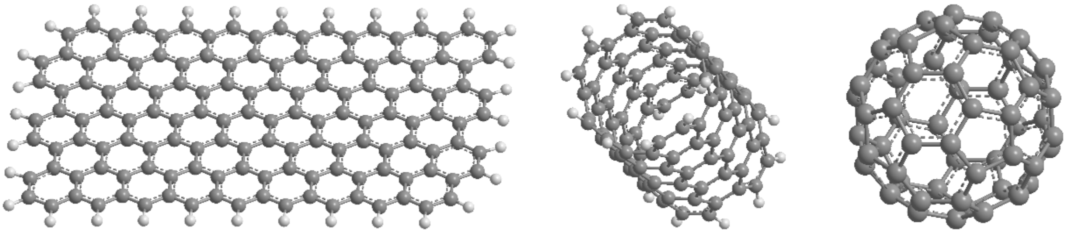

(**a**) (**b**) (**c**)

**Figure S1.** Schematic of carbon-based nanomaterial structure. (**a**) GR. (**b**) CNT. (**c**) $C_{60}$.

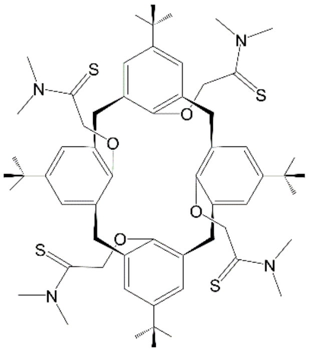

**Figure S2.** The structure of the lead ionophore IV, 4-tert-butylcalix [4] arene-tetrakis (N,N-dimethylthioacetamide).

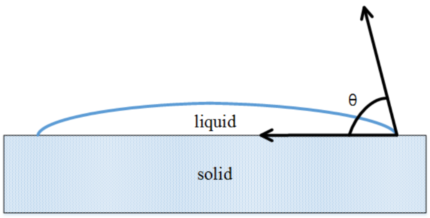


**Figure S3.** Schematic of a sessile-drop contact angle system

**Table S1.** Comparison of GC/GR/$Pb^{2+}$-ISEs with different volume of $Pb^{2+}$-ISM characteristics.

| Volume (μL) | Slope (mV/decade) | LDL (mol/L) | Response time (s) |
|---|---|---|---|
| 10 | 24.7 | $9.1\times10^{-8}$ | 50.7 |
| 15 | 25.2 | $6.9\times10^{-8}$ | 42.0 |

| | | | |
|---|---|---|---|
| 20 | 26.8 | $3.4\times10^{-8}$ | 42.6 |
| 25 | 25.9 | $6.4\times10^{-8}$ | 49.1 |
| 30 | 24.9 | $1.0\times10^{-7}$ | 53.5 |

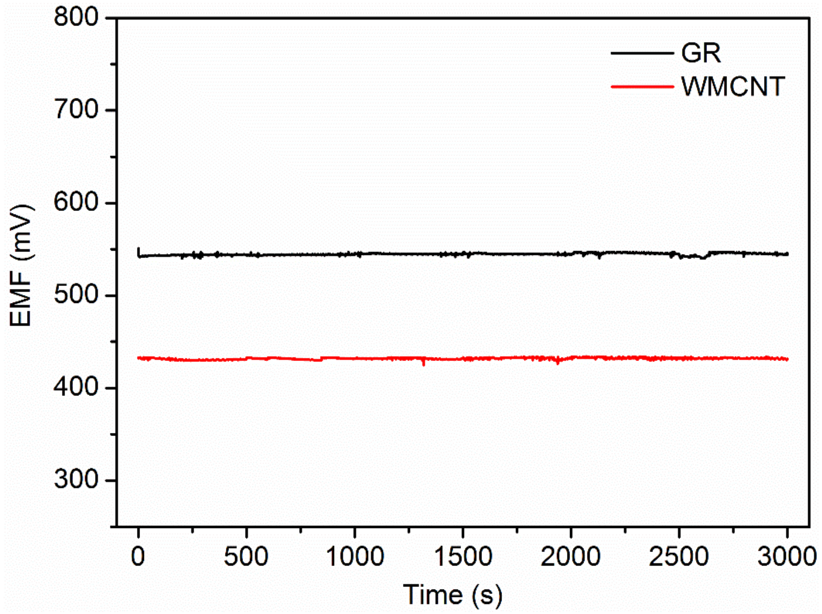


**Figure S4.** EMF of GC/GR/$Pb^{2+}$-ISE and GC/MWCNT/$Pb^{2+}$-ISE in $10^{-5}$ mol/L lead solution.
